# 3,5,4′-trihydroxy-6,7,3′-trimethoxyflavone protects against beta amyloid-induced neurotoxicity through antioxidative activity and interference with cell signaling

**DOI:** 10.1186/s12906-017-1840-y

**Published:** 2017-06-23

**Authors:** Alona Telerman, Rivka Ofir, Yoel Kashman, Anat Elmann

**Affiliations:** 1 Department of Food Quality and Safety, Volcani Center, Agricultural Research Organization, POB 15159, 7528809 Rishon Lezion, Israel; 2 Dead Sea and Arava Science Center, Central Arava Branch, 8682500 Merkaz Sapir, Israel; 3 Raymond and Beverly Sackler Faculty of Exact Sciences, School of Chemistry, Tel Aviv University, Ramat Aviv, 69978 Tel Aviv, Israel

**Keywords:** 3,5,4′-trihydroxy-6,7,3′-trimethoxyflavone, Alzheimer’s disease, Neurons, Flavonoids, Amyloid beta, *Achillea fragrantissima*, Mitogen-activated protein kinases

## Abstract

**Background:**

Alzheimer’s disease is a neurodegenerative disease, characterized by progressive decline in memory and cognitive functions, that results from loss of neurons in the brain. Amyloid beta (Aβ) protein and oxidative stress are major contributors to Alzheimer’s disease, therefore, protecting neuronal cells against Aβ-induced toxicity and oxidative stress might form an effective approach for treatment of this disease. 3,5,4′-trihydroxy-6,7,3′-trimethoxyflavone (TTF) is a flavonoid we have purified from the plant *Achillea fragrantissima*; and the present study examined, for the first time, the effects of this compound on Aβ-toxicity to neuronal cells.

**Methods:**

Various chromatographic techniques were used to isolate TTF from the plant *Achillea fragrantissima*, and an N2a neuroblastoma cell line was used to study its activities. The cellular levels of total and phosphorylated stress-activated protein kinase/c-Jun N-terminal kinase (SAPK/JNK) and of total and phosphorylated extracellular signal-regulated kinase (ERK 1/2) were determined by enzyme-linked immunosorbent assay (ELISA). Intracellular reactive oxygen species (ROS) levels were measured by using 2′,7′-dichlorofluorescein diacetate (DCF-DA). Cytotoxicity and cell viability were assessed by using lactate dehydrogenase (LDH) activity in cell-conditioned media, or by crystal violet cell staining, respectively.

**Results:**

TTF prevented the Aβ-induced death of neurons and attenuated the intracellular accumulation of ROS following treatment of these cells with Aβ. TTF also inhibited the Aβ-induced phosphorylation of the signaling proteins SAPK/JNK and ERK 1/2, which belong to the mitogen-activated protein kinase (MAPK) family.

**Conclusion:**

TTF should be studied further as a potential therapeutic means for the treatment of Alzheimer’s disease.

## Background

Alzheimer’s disease (AD), the most common form of dementia in adults, is characterized by widespread loss of neurons in the brain, which results in progressive memory loss and cognitive decline. AD is characterized by intraneuronal neurofibrillary tangles (aggregates of cytoskeletal hyperphosphorylated Tau protein) and extraneuronal senile plaques, formed mainly by aggregated amyloid beta (Aβ) peptides [1]. Aβ is a 39- to 43-amino acid peptide derived from the cleavage of amyloid precursor protein (APP), and it is involved in the pathogenesis of AD through several different mechanisms, including oxidative stress, microglial activation, neuronal dysfunction, and neuronal cell death [2, 3]. Thus, protecting neuronal cells against Aβ-induced cytotoxicity might form an effective approach to therapeutic treatment of Alzheimer’s disease.

An increasing body of evidence shows the therapeutic potential of phytochemicals against various diseases, including AD [4, 5]. Flavonoids are phytochemicals that exhibit a broad range of biological functions, including anti-inflammatory, antioxidative and neuroprotective activities [6–11]. In light of these activities, they seem to be promising candidates for development as drugs for treatment of neurodegenerative diseases [8, 12, 13].

In a previous study, we have shown that 3,5,4′-trihydroxy-6,7,3′-trimethoxyflavone (TTF), a flavonoid that we have isolated from *Achillea fragrantissima*, prevented the hydrogen peroxide (H_2_O_2_)-induced death of astrocytes, and inhibited the phosphorylation of cell-signaling proteins that belong to the mitogen-activated protein kinase (MAPK) family [14]. TTF also scavenges free radicals and mitigates intracellular accumulation of ROS following treatment of these cells with H_2_O_2_ or with the peroxyl radicals-generating molecule 2,2′-azobis(amidinopropane) (ABAP) [14]; it is characterized by a relatively low polarity and low molecular weight of 360.3, therefore it might cross the blood-brain barrier and could be used for further investigations in cellular and animal models of AD.

In the present study, we aimed to determine whether TTF could counteract Aβ toxicity in N2a neuroblastoma cells, and to elucidate the molecular mechanisms involved.

## Methods

### Chemicals and reagents

Aβ_25–35_, crystal violet, and 2′,7′-dichlorofluorescein diacetate (DCF-DA) were purchased from Sigma Chemical Co. (St Louis, MO, USA); Dulbecco’s modified Eagle’s medium (DMEM) and Opti-MEM were purchased from Gibco (Paisley, UK); glutamine, antibiotics (10,000 IU/mL penicillin and 10,000 μg/mL streptomycin), fetal bovine serum (FBS) and trypin-EDTA were purchased from Biological Industries (Beit Haemek, Israel); dimethyl sulfoxide (DMSO) was obtained from Applichem (Darmstadt, Germany).

### Plant material

The aerial parts of *A. fragrantissima* were collected in the Arava Valley, and the voucher specimens have been kept and authenticated as part of the Arava Rift Valley Plant Collection under accession code AVPC0040.

### Extraction and isolation

Isolation of TTF was carried out as previously described [14]. Briefly: the sun-dried *A. fragrantissima* plant was homogenized and extracted with ethyl acetate twice and with ethyl acetate:methanol (9:1) once. The organic extracts were combined and evaporated. The latter residue was chromatographed on a Sephadex LH-20 column that was eluted with methanol: CH_2_Cl_2_ (1:1). A fraction yielded by the Sephadex LH-20 column, which was monitored by thin layer chromatography (TLC) and ^1^H–nuclear magnetic resonance (NMR) and that protected astrocytes from H_2_O_2_-induced cell death, was further purified by repeated chromatography over silica gel, with hexane that contained increasing proportions of ethyl acetate used as eluent. The active compound was afforded by elution with 50% ethyl acetate in hexane.

### Treatment of neuronal cells

The original medium was aspirated from the cells and replaced with fresh medium. Fresh dilutions of TTF, first in DMSO and then in the growth medium, were prepared from stock solution just prior to each experiment, and were used immediately. The final concentration of DMSO in the medium was 0.2%. The Aβ_25–35_ peptide was dissolved in DDW and incubated at 37 °C for 48 h. Fresh dilutions of Aβ in the growth medium were prepared just prior to each experiment and were used immediately. Each treatment was performed in replicates.

### Determination of cytotoxicity and cell viability

*Cytotoxicity* **–** N2a cells were grown in a medium containing 43% DMEM (high glucose), 50% Opti-MEM, 5% FBS, 2 mM glutamine, penicillin at 100 U/mL, and streptomycin at 100 μg/mL. The cells were re-plated in 96-well plates at a density of 5 × 10^3^ cells/well, in a similar medium; Aβ and/or TTF were added concomitantly 24 h later, and cytotoxicity was determined 20 h later with a commercial colorimetric assay (Roche Applied Science, Germany) based on the measurement of lactate dehydrogenase (LDH) activity released into the incubation medium from the cytosol of damaged cells. The absorbance was measured at 492 nm in a Synergy2 multi-detection microplate reader (BioTek Instruments, Inc., Winooski, VT, USA). The percentage of cytotoxicity was calculated according to:

$$ Cytotoxicity\ \left(\%\right)=\frac{\left({A}_{treated\  cells}-{A}_{untreated\  cells}\right)\times 100}{A_{Triton- X\  treated\  cells}-{A}_{untreated\  cells}} $$

in which the term “A_Triton-x treated cells_” is the maximum releasable LDH from the cells.

*Cell viability* – N2a cells were grown and treated as in the cytotoxicity assay, except that replating was at a density of 5 × 10^3^ cells/well. Cell viability was determined by a modification of the crystal violet assay [15], as follows. At the end of their treatments the cells were fixed with 150 μL of 5% (*v*/v) formaldehyde in PBS for 15 min at room temperature. The plates were washed by immersion in deionized water, dried and stained for 15 min with 150 μL of a 1% crystal violet solution. Following aspiration of the crystal violet solution the plates were washed with deionized water and dried, and the bound dye was solubilized with 150 μL of 33% aqueous solution of glacial acetic acid. The optical density was measured at 540 nm, by comparison with a 690-nm reference filter, in a Synergy2 multi-detection microplate reader (BioTek Instruments, Inc., Winooski, VT, USA).

### Evaluation of intracellular ROS levels

Intracellular ROS levels were detected by using the non-fluorescent cell-permeating compound, 2′,7′-dichlorofluorescein diacetate (DCF-DA). The N2a cells were plated onto 96-well plates at 10,000 cells per 0.2-mL well, in 43% DMEM (high glucose), 50% Opti-MEM, 1% FBS, 2 mM glutamine, penicillin at 100 U/mL, and streptomycin at 100 mg/mL. After 24 h the cells were treated with 20 μM DCF-DA for 30 min at 37 °C. Following incubation with DCF-DA, the cultures were rinsed twice with PBS, which was then replaced with fresh medium. The*.* ROS levels at time zero were evaluated according to fluorescence in a Synergy2 multi-detection microplate reader (BioTek Instruments, Inc., Winooski, VT, USA) with excitation at 485 nm and emission at 520 nm. The cells were then treated with TTF and Aβ, and the ROS levels were measured at the indicated time points.

The percentage of ROS levels was calculated according to:

$$ ROS\  levels\ \left(\%\right)=\frac{\left({F}_{TTF\& A\beta - treated\  cells}-{F}_{untreated\  cells}\right)\times 100}{F_{A\beta - treated\  cells}-{F}_{untreated\  cells}} $$

in which F is the fluorescence.

### Enzyme-linked immunosorbent assays (ELISA) for total and phosphoSAPK/JNK, and total and phosphoERK (phospho-p44/42 MAPK)

N2a cells were treated with TTF concomitantly with addition of Aβ_25–35_. The cells were lysed after 40 or 30 min, for SAPK/JNK or ERK 1/2, respectively, in lysis buffer that was part of the PathScan Sandwich ELISA kit (Cell Signaling Technology, Danvers, MA, USA) according to the manufacturer’s protocol. Protein concentrations in cell lysates were determined with Bradford reagent (Bio-Rad, Hercules, CA, USA), and equal amounts of proteins were subjected to ELISA. To measure the amounts of total and of phosphoSAPK/JNK in cell lysates of the N2a cells, ELISA was performed according to the manufacturer’s protocols with the PathScan total SAPK/JNK sandwich ELISA kit and the PathScan phosphoSAPK/JNK (Thr183/Tyr185) sandwich ELISA kit, respectively (both kits from Cell Signaling Technology, Danvers, MA, USA). To measure the amounts of total and phosphoERK 1/2, i.e., phospho-p44/42 MAPK, in cell lysates, ELISA was performed according to the manufacturer’s protocol with Cell Signaling Technology’s PathScan total p44/42 MAPK (ERK 1/2) sandwich ELISA kit and the PathScan phospho-p44/42 MAPK (Thr202/Tyr204) sandwich ELISA kit, respectively. The optical density was measured at 450 nm with a Synergy2 multi-detection microplate reader (BioTek Instruments, Inc., Winooski, VT, USA).

### Statistical analysis

The results were subjected to one-way ANOVA followed by Tukey-Kramer multiple comparison tests, by means of Graph Pad InStat 3 for Windows (GraphPad Software, San Diego, CA, USA).

## Results

### **Protective effect of TTF against A**β_**25–35**_**-induced neuronal cell death**

**Fig. 1:**
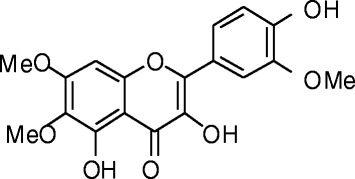
Chemical structure of 3,5,4′-trihydroxy-6,7,3′-trimethoxyflavone (TTF)

**Fig. 2:**
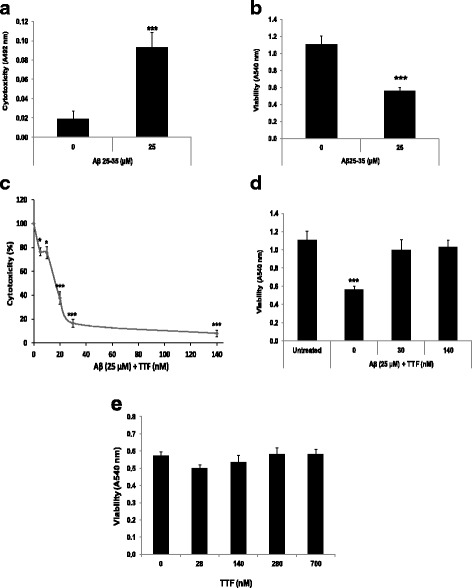
TTF protects neuronal cells from Aβ-induced cell death*.* Neuronal cells were treated with Aβ_25–35_ at 25 μM, and cell death was determined 20 h later by **(a)** the LDH and **(b)** the crystal violet methods. Neuronal cells were treated with various concentrations of TTF, Aβ was added and cell death was determined 20 h later by **(c)** the LDH and **(d)** the crystal violet methods. The results are means ± SEM of two experiments (*n* = 16); *** P < 0.001, for comparison of treated with untreated cells. The maximal LDH release after disruption of cells by Triton x-100 was A492 = 0.61 ± 0.04 as measured in two experiments (*n* = 5). **(e)** Cells were treated with TTF alone, at various concentrations, and cell viability was determined 20 h later by the crystal violet method. The results are means ± SEM of two experiments (*n* = 16). *** P < 0.001, for comparison with cells that were treated with Aβ alone

Phytochemicals that can protect neuronal cells against Aβ toxicity and oxidative stress may assist in coping with Alzheimer’s disease. In order to assess the ability of TTF (The structure of TTF is presented in Fig. 1) to counteract Aβ toxicity in N2a neuroblastoma cells, we have used the Aβ_25–35_ peptide, which represents the neurotoxic fragments of Aβ_1–40_ and Aβ_1–42_, and is used in cell models to mimic their toxicity. Exposure of neuronal cells to Aβ_25–35_ resulted in their death after 20 h, as reflected in a fivefold increase in the LDH assay (Fig. 2a), and a 55% reduction in the crystal violet assay (Fig. 2b). To assess the ability of TTF to protect neuronal cells against Aβ_25–35_, and to determine the optimal concentrations of TTF required to induce a protective effect, cells were treated with Aβ_25–35_ at 25 μM and with various concentrations of TTF. Cytotoxicity and viability were determined after 20 h by means of the LDH assay (Fig. 2c) and the crystal violet assay (Fig. 2d), respectively. Our results show that TTF exhibited a protective effect against Aβ_25–35_-induced cell death, with maximal efficacy (96% protection) at a concentration of 140 nM (Fig. 2c, d). It should be noted that at all concentrations tested, up to 700 nM, the crystal violet assay did not show TTF alone to be cytotoxic to neuronal cells (Fig. 2e).

### **Inhibitory effect of TTF on A**β_**25–35**_**-induced generation of ROS**

**Fig. 3:**
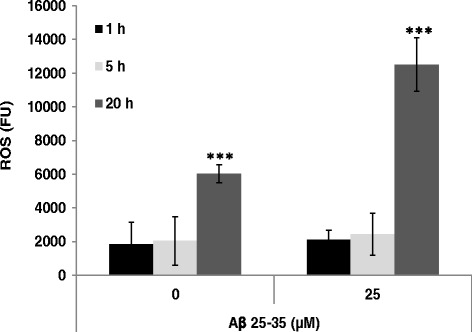
Aβ-induced elevation in ROS levels in N2a cells. Intracellular ROS levels (Fluorescence units, FU) were measured 1, 5, and 20 h after treatment. The results are the mean ± SEM of one experiment (*n* = 8)

The role of free radicals in AD has been reported in many studies [16]. Moreover, it has been shown that Aβ induced generation of reactive oxygen species (ROS), leading to neuronal death [3]. To investigate the effect of TTF on ROS levels, which are elevated in response to Aβ treatment, the levels of intracellular ROS were determined. Treatment of cells with Aβ_25–35_ for 20 h resulted in a twofold increase in intracellular ROS levels (Fig. 3), but no significant elevation in ROS levels was observed one or five hours after stimulation (Fig. 3). We therefore tested the possibility that TTF could protect neuronal cells from Aβ_25–35_-induced cell death by inhibiting the Aβ_25–35_-induced production of ROS. Cells were treated with various concentrations of TTF, simultaneously with application of Aβ_25–35_. Changes in intracellular levels of ROS were detected with the ROS indicator DCF-DA, and ROS formation was determined by examining fluorescence after 20 h. Our results show that treatment with TTF at a concentration of 25 nM – similar to that used to protect cells from Aβ_25–35_-induced cell death – inhibited the intracellular levels of Aβ_25–35_-induced ROS by 60% (Table 1).

**Table 1:** TTF attenuates Aβ induced ROS levels in N2a cells

TTF (nM)	ROS (%)
0	100
2	77 ± 9*
12	48 ± 6***
25	39 ± 6***

Cells were treated for 20 h with Aβ at 25 μM and with various concentrations of TTF. The results represent the means ± SEM of three experiments (*n* = 24). *p < 0.05, ***p < 0.001, for comparison with cells that were treated with A_β_ alone

### **Inhibitory effect of TTF on A**β**-induced phosphorylation of SAPK/JNK and ERK 1/2 in N2a cells**

Mitogen-activated protein kinases (MAPKs) – a family of serine/threonine protein kinases – which initiate many cellular responses to external stress signals, were shown to be modulated by flavonoids [12, 17]. Enhanced activation of SAPK/JNK and ERK 1/2, which belong to the MAPK cascade, was observed in AD-affected brains [18–20]. Moreover, Aβ has been reported to stimulate SAPK/JNK and ERK 1/2 [21–24]. Therefore, we determined the effect of TTF on Aβ_25–35_-induced phosphorylation of SAPK/JNK and ERK 1/2.

**Fig. 4:**
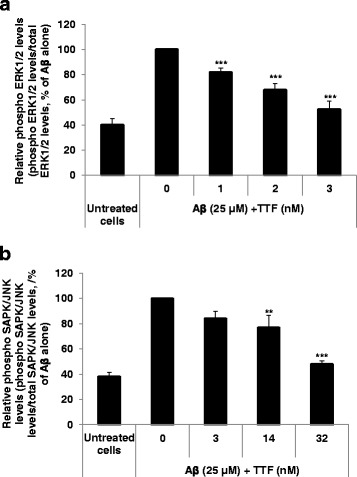
TTF suppresses Aβ-induced phosphorylation of SAPK/JNK, and ERK 1/2 in N2a cells. Cells were treated concomitantly with 25 μM of Aβ and TTF for 30 min (ERK 1/2) or 40 min (SAPK/JNK). Cells were extracted and the levels of phosphorylated and total ERK 1/2 (**a**) and SAPK/JNK (**b**) were determined with specific ELISA kits. The levels of each of the phosphorylated proteins were normalized to the levels of the total amount of the related proteins, and are presented as means ± SEM of two experiments (*n* = 4) for SAPK/JNK, and of three experiments (*n* = 6) for ERK 1/2. **p < 0.01, ***p < 0.001, for comparison with cells that were treated with Aβ only

As can be seen in Fig. 4, treatment with Aβ_25–35_ increased cellular levels of phosphorylated SAPK/JNK and ERK 1/2 by 2.5-fold. Treatment of cells with TTF at 3 and 32 nM inhibited the Aβ_25–35_-induced phosphorylation of ERK 1/2 and SAPK/JNK, respectively, by 84 and 60%, respectively (Figs 4a and b, respectively). The total amounts of these proteins in the cells were not affected by TTF. It should be noted that TTF was more effective in inhibiting phosphorylation of ERK 1/2 than that of SAPK/JNK: at 3 nM it inhibited induced phosphorylation of ERK 1/2 by 84%, whereas at this concentration it inhibited that of SAPK/JNK by only 26%. These results suggest that inhibition of Aβ-induced phosphorylation of SAPK/JNK and ERK 1/2 is part of the mechanism by which TTF protects cells against Aβ_25–35_ toxicity.

## Discussion

In the present study we showed, for the first time, that TTF, a natural flavonoid that we isolated from *A. fragrantissima* [14], could protect neuronal cells against Aβ-induced cell death, and that it inhibited phosphorylation of MAP kinases and attenuated the intracellular accumulation of ROS following treatment with Aβ. To the best of our knowledge, this is the first study that investigated the effects of TTF on neuronal cells and on Aβ-induced cytotoxicity.

Drugs currently used for treating AD improve patients’ functions symptomatically, but do not modify the disease mechanism; thus, development of new and more effective drugs is required. Herbs and medicinal plants have been demonstrated in animal and cellular models to exhibit various interventions against multiple targets related to AD, including anti-cholinesterase activity, anti-amyloid, anti-oxidant and anti-inflammatory, and therefore might affect disease progression [25–27]; thus, they might affect disease mechanisms. Nutraceuticals that can protect neuronal cells from Aβ toxicity and oxidative stress are potential candidates for the treatment of Alzheimer’s disease. It previously has been suggested that new strategies based on memantine combined with antioxidants, could provide a multitargeted therapy to enhance neuronal protection and prevent disease progression [28]. The antioxidant activities of TTF demonstrated in neuronal cells in the present study, and in astrocytes in our previous study [14] indicate that TTF is a potential component of combined therapies.

Most flavonoids are metabolized in the gastro-intestinal tract and liver and are absorbed into the bloodstream; and some of them were shown to cross the blood/brain barrier (BBB) into the central nervous system [29–33]. Flavonoids were shown to have neuroprotective effects, to impact neuronal function, to modulate neurotransmission, and to improve synaptic plasticity and cognition. Moreover, although astrocytes were proposed to mediate the actions of flavonoids in the brain, little is known about their cellular targets [32]. Aβ-induced neurotoxicity might be mediated through several Aβ-binding proteins that were identified on neuron plasma membranes. They include: N-methyl-D-aspartate receptor (NMDAR); receptor for advanced glycation end products (RAGE); α7-nicotinic acetylcholine receptor (α7 nAChR); ephrin type B receptor 2; cellular prion protein (PrPc); immunoglobulin G Fc gamma receptor IIb (FcgRIIb); and paired immunoglobulin-like receptor B (PirB) [34]. Accordingly, TTF might manifest its protective effects by antagonizing these receptors. Our results that relate to inhibition of MAPK are compatible with other findings that showed flavonoids to modulate MAP-kinase signaling, or to directly bind to some protein kinases [35, 36].

## Conclusions

Taken together, our findings suggest that by interfering with Aβ-induced signaling events and inhibiting elevation of intracellular ROS, TTF protects cells against Aβ-toxicity. In light of our previous results in astrocytes and our present results in neuronal cells, as well as the results of oral safety studies of extracts prepared from *A. fragrantissima* [37], further studies should be conducted, to substantiate the potential of TTF for therapeutic treatment of AD.

## Abbreviations

*A. fragrantissima*: *Achillea fragrantissima* (Forssk) Sch. Bip
AD: Alzheimer’s disease
Aβ: Amyloid beta
DCF-DA: 2′,7′-dichlorofluorescein diacetate
ERK: Extracellular signal regulated kinase
MAPK: Mitogen-activated protein kinase
ROS: Reactive oxygen species
SAPK/JNK: Stress-activated protein kinase /c-Jun N-terminal kinase
TTF: 3,5,4′-trihydroxy-6,7,3′-trimethoxyflavone
